# Quality of reporting health behaviors for multiple sclerosis (QuoRH‐MS): A scoping review to inform intervention planning and improve consistency of reporting

**DOI:** 10.1002/brb3.3635

**Published:** 2024-08-15

**Authors:** Yasmine Probst, Emily Kinnane

**Affiliations:** ^1^ School of Medical, Indigenous and Health Sciences University of Wollongong Wollongong New South Wales Australia

**Keywords:** health behavior, neurodegenerative disease, quality, review

## Abstract

**Background:**

Multiple sclerosis (MS) is a neurological condition that necessitates a multidisciplinary approach to aid those living with MS in managing their disease. Health behavior, or lifestyle modification, is an emerging approach to MS self‐management. MS researchers utilize measurement tools to ensure that interventions are best suited to the outcomes, thereby potentially influencing practice. The aim of this study was to investigate which tools are being used for health behavior management studies in people living with MS and develop an aid for tool selection.

**Methods:**

A scoping review guided by the PRISMA‐Sc checklist and the JBI manual for evidence synthesis was employed with a systematic search strategy executed across four scientific databases: Medline, PubMed, CINAHL, and Cochrane Libraries. The types of assessment tools used were extracted from the included studies. Each tool was categorized into the health behavior intervention discipline (nutrition, exercise, and psychology) and then subcategorized by the tool's purpose. The frequency of use was determined for each tool. Reporting of validation of the assessment tools were collated to inform a tool selection checklist.

**Results:**

The review identified a total of 248 tools (12 nutrition, 55 exercise, and 119 psychology unique reports) from 166 studies. Seventy‐seven multidimensional tools were identified including measures of quality of life, fatigue, and functional scales. Only 88 studies (53%) referred to the validity of the tools. The most commonly reported tools were the dietary habits questionnaire (*n* = 4, nutrition), 6‐minute walk test (*n* = 17, exercise), Symbol Digits and Modalities Test, and Hospital Anxiety and Depression Scale (*n* = 15 each, psychology) with the Expanded Disability Status Scale reported 43 times.

**Conclusion:**

Evidence from interventions may inform practice for health professionals. This review provides insights into the range of tools reported across health behavior intervention studies for MS and offers a guide toward more consistent reporting of study methods.

## Graphical abstract

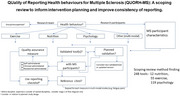

## 1 INTRODUCTION

Multiple sclerosis (MS) is a chronic neurodegenerative condition that is variable in its presentation, globally affecting 3.3 million people (Walton et al., 2020). Reduced conduction signaling along the nerves can cause people living with MS (plwMS) to experience a range of symptoms including disruptions to gait, balance, and coordination, increased fatigue, and spasticity (White & Dressendorfer, 2004). Disease management is focused on reducing the time to commencing disease‐modifying therapies, though nonpharmacological symptom management of MS is increasingly recognized.

Lifestyle management programs for plwMS aim to assist with the self‐management of activities of daily living to support behavior change while accommodating symptoms of the disease (Roessler et al., 2004). Programs recognize the unique nature of the disease course and are often tailored to needs and experiences of plwMS. A recent meta‐analysis reported that lifestyle programs often comprise multicomponent self‐management approaches using multimodal methods of delivery and principles of cognitive behavioral therapy, finding that these elements improved the reported quality of life of participants (Wills & Probst, 2022). The review also reported a high level of heterogeneity in the programs. Lifestyle management programs for MS may include activities for skill development through exercise, stress management strategies, meal planning and food choice, and time management strategies or schedules (Roessler et al., 2004).

While behavior change is often considered separate from pharmaceutical therapies, many lifestyle programs do consider medication management. Disease‐modifying therapies are a first‐line therapy for MS and play a crucial role in disability progression and symptom management (White & Dressendorfer, 2004). Polypharmacy is commonly reported due to comorbidities, symptom management, and secondary pathologies (Frahm et al., 2020). Therefore, lifestyle management programs need to consider strategies for disease‐modifying therapies as part of health behavior interventions.

Studies have demonstrated the role of exercise interventions through rehabilitation programs for plwMS to improve quality of life and enable activities of daily living (Motl et al., 2017). Planning for best‐practice assessments prior to an exercise intervention is crucial to accommodate for the unique presentation of symptoms and disability, and preserving and/or increasing physical function is a known benefit of exercise for MS (Learmonth & Motl, 2016). A recent study by Marck et al. investigated the types and duration of exercise for plwMS, identifying strength and aerobic training as the most used forms of exercise intervention (Marck et al., 2022). However insufficient evidence scope and quality alongside a poor understanding of the mechanisms for MS and exercise were identified as limitations (Motl et al., 2017). Interestingly, previous studies that have investigated exercise and its effect on MS were heavily focused on quality of life rather than the types of exercise that are most beneficial (Alphonsus et al., 2019).

Nutrition behaviors are increasingly recognized for symptom management of MS (Beckett et al., 2019). While a specific diet has not been identified (Beckett et al., 2019), clinicians are encouraged to recommend a healthy and balanced diet (Bagur et al., 2017) to their clients. A recent study also suggested that while a diet should not replace pharmaceutical management, it has a compounding effect and may allow for more efficient symptom management (Stoiloudis et al., 2022). Nutrition may have a role in reducing physical disability including mobility restrictions and issues related to swallowing and has an impact on the preparation of food as well as the ability to eat food due to fatigue, cognitive decline, and/or depression. Nutrition behaviors also play a role in relation to malnutrition and nutrient imbalance leading to deficiency and increased metabolic risk. For example, elevated blood pressure and blood glucose levels, more often observed in plwMS in comparison to the general population (Esposito et al., 2018), are managed by diet. While the prevalence of malnutrition is not known, previous studies have revealed it is more frequently identified in plwMS than in those living with other chronic conditions (Esposito et al., 2018).

A combination of exercise and nutrition behavior change strategies in parallel with disease‐modifying therapy has been shown to help with the physical functioning in plwMS. However, as the disease is unpredictable, strategies to support these practices are required. Artemiadis et al. identified that while no coping strategy is more effective or of greater benefit than the other, strategies that increase an individual's perceived control can cause the least distress. Strategies may include relaxation training and cognitive behavioral therapy, including self‐monitoring of stress, problem‐solving, and cognitive restructuring (Reynard et al., 2014). Like exercise and nutrition, stress management aims to improve functional ability. To justify the use of stress management strategies, psychological assessments may be conducted to identify strategies with the most benefit (Groth‐Marnat & Wright, 2016).

The management of MS is complex and relies on health professionals having access to evidence‐based guidance to inform practice. Therefore, the aim of this study was to collate the assessment tools used for lifestyle management studies with plwMS to inform the development of an approach to guide tool selection.

## 2 METHODS

A methodological scoping review was used to answer the question “What assessment tools are being used for lifestyle management studies for people living with multiple sclerosis?” A scoping review aims to determine the breadth of an area and is often used to identify gaps and inform practice around key concept areas (Munn et al., 2018). This review was registered with the open science framework on March 18, 2022 (https://osf.io/jwfd4/), and follows the JBI manual for evidence synthesis (Peters et al., 2020) reported in line with the PRISMA ScR checklist (Tricco et al., 2018).

### 2.1 Search strategy

Key concepts for the review were identified as “multiple sclerosis,” “lifestyle management programs,” “exercise,” “nutrition,” “psychology,” and “medication.” Search terms using keywords and MeSH terms were formulated to create the search strategy (Table S1), which was applied to the databases PubMed, Medline, CINAHL, and Cochrane Libraries, as recommended by the *Cochrane Handbook for Systematic Reviews of Interventions* (Higgins, 2022). Pilot searches with each database tested the feasibility of the search, and sentinel articles were used to confirm the final search with adjustments made for each database as required. No date limits were applied to the search, given no prior reviews or similar studies of this nature have been conducted for multiple sclerosis research. This is also aligned with the recommendations of the Cochrane handbook.

### 2.2 Eligibility

Records generated from the searches were collated and managed using Covidence Review Software (Veritas Health Innovation, Melbourne, Australia; available at https://www.covidence.org.) and assessed independently against the inclusion criteria by two researchers Y.P. and E.K. Where conflicts occurred, the researchers discussed the outcome to reach a consensus. Included studies included adults diagnosed with MS who implemented a lifestyle intervention (including exercise, nutrition, and/or psychological components) for symptom management or to slow disease progression (including disease management). The types of tools used to assess each domain of lifestyle were the primary outcomes. Studies that used other tools needed to include one of the three lifestyle domains to be eligible. For example, studies that addressed medication and other factors, for example, symptom management only, were excluded, while studies that included medication and resistance exercise were included. Excluded studies also included conference abstracts, protocol papers, pediatric populations, or those with pregnant females as well as animal studies, in vitro or in vivo studies, studies of MS risk, and studies that used complementary and alternative medicines.

Records from all countries and published in all languages were included. Google Translate (https://translate.google.com/) was used for studies not published in the English language. Review articles were excluded; however, reference lists were manually screened against the inclusion criteria for additional studies.

### 2.3 Data extraction

Eligible records were examined with author, year of publication, country of study, study design, population characteristics (description, age, disability), sample size, and lifestyle domain addressed, and the tools used to measure each domain extracted and collated into a summary table. Where the authors mentioned validation of any of the tools used in the methods of the study, this was also collated. Each lifestyle domain was subcategorized to the discipline areas of nutrition, exercise, or psychology. Subcategories were defined by the *Manual of Dietetic Practice* (2019) for nutrition (Gandy, 2019), the *Exercise and Sports Science Australia Guidelines* (Coombes & Skinner, 2022) for exercise, and the *Handbook of Psychological Assessment* (Groth‐Marnat & Wright, 2016) for psychology (Table S2). The frequency of use of each tool was calculated based on the number of times a single tool appeared across the included studies and each of the domains of tool categorization.

### 2.4 Checklist development and testing

A checklist was developed from the included studies focusing on the need for validation of the tools with an MS population. Using the validity reporting from the studies, patterns in the outcomes were used to inform a flow chart and checklist that can be used to guide researchers in future planning of studies for MS. The checklist was tested for face validity using a random sample of included studies from the review.

## 3 RESULTS

The search strategy generated a total of 26,962 records. After the initial screening, 579 records were eligible and the final screening resulted in 166 studies included in the review (Figure S1). There were 14 studies for the nutrition domain, 71 that included exercise, and 103 studies that included psychology many targeting more than 1 domain. Only four studies included all domains while 19 studies included two domains of either nutrition and exercise or exercise and psychology. No studies explicitly targeted nutrition and psychology (Table 1). In total, 12 tools were classified as other with three related to medication, two to anthropometry, eight impact scales, four pain scales, 12 for assessing fatigue and sleep, 17 for health and quality of life, seven functional assessments, and nine acceptance and perception of disease tools. Sixty‐one studies had a randomized controlled trial study design, 11 were observational studies, and 49 were cross‐sectional analyses. Table 2 represents the differences in the frequency of use of the different tool types indicating a substantial difference for the multimodal tool types with impact scales, namely the Expanded Disability Status Scale (EDSS), used most frequently (43 times) across its 124 included studies. The EDSS was often used to confirm participant eligibility based on a predetermined level of disability.

**TABLE 1:** Summary table for all included studies.

Ref.	Country	Study design	*N* =	Population at commencement			Nutrition	Exercise	Psychology	Medication	Other
				Diagnosis	Age (year), EDSS						
Agland et al. (2018)	Australia	Waitlisted randomized trial	100	Any diagnosis	18–70	–			✔		✔
Alghwiri et al. (2020)	Iran	RCT	60	MMSE ≥ 24	≥18	<6.5		✔	✔		✔
Amato et al. (2021)	Italy	Quasi experimental	8	McD, no relapse 12 months	20–55	1.5–3.5	✔	✔	✔		✔
Anens et al. (2014)	Sweden	Cross‐sectional	287	From Swedish MS Registry	18–80	–		✔			✔
Anens et al. (2017)	Sweden	Cross‐sectional	287	From Swedish MS Registry	18–80	–		✔			✔
Artemiadis et al. (2012)	Greece	RCT	73	RRMS, Poser or McD, DMT	18–65	<4			✔		✔
Azimian et al. (2021)	Iran	RCT	71	Referred to the hospital, no steroid DMT	20–50	<5		✔	✔		✔
Sadeghi Bahmani et al. (2019)	Iran	RCT	92	Females, McD	18–65	<6			✔		✔
Bakshi et al. (2000)	United States	Cross‐sectional	71	Clinically definite	18–60	–			✔		✔
Banitalebi et al. (2020)	Iran	RCT	94	Females, RRMS	18–50	–		✔			
Bassi et al. (2014)	Italy	Cross‐sectional	70	McD, ≥3 years with carer	40 (mean)	<8			✔		✔
Bijani et al. (2022)	Iran	RCT	90	McD, ≥6 months	20–55	–			✔		✔
Bogosian et al. (2016)	England	Waitlisted pilot RCT + qualitative interviews	40	RRMS or SPMS, > 3 GHQ‐12, < 20 TICS‐m	–	–			✔		✔
Boogar et al. (2018)	Iran	Cross‐sectional	193	RRMS, Milo and Miller criteria, DASS‐21 ≥20, MMSE ≥24	21–62	–			✔		✔
Brenton and Goldman (2016)	United States	Cross‐sectional cohort	199	MS patients from Multiple Sclerosis Clinic, all types	–	–		✔			✔
Brenton et al. (2022)	United States	Clinical trial	64	RRMS, McD, stable > 6 months	12–55	≤6	✔	✔	✔		✔
Canning and Hicks (2020)	Canada	RCT	91	MS diagnosis	18–64	1–7		✔			
Carletto et al. (2017)	Italy	RCT	90	Patients from University Hospital of Orbassano, McD, no relapse > 3 months, steroid DMT > 30 days, BDI‐II > 13	18–65	<6.5			✔		✔
Carvalho and Sá (2012)	Portugal	Observational, retrospective	26	RRMS McD, first DMT 1996–1999, maintained treatment to 2012	≥18	–				✔	✔
Cavalera et al. (2019)	Italy	RCT	139	RRMS or SPMS, no DMT change > 3 months, no relapses or steroid > 4 weeks	≥18				✔		✔
(Christopoulos et al., 2020)	Greece	Cross sectional, case‐matched	80	McD	43.28 (mean)	–			✔		✔
(Cohen et al., 2019)	United States	RCT	60	McD, T25FW 8–45 s, stable DMT	18–70	≤6.5		✔	✔		✔
Coote et al. (2017)	Ireland	RCT	92	From MS Society of Ireland, McD or Poser, sedentary lifestyle	≥18	0–3^a^		✔	✔		✔
Crescentini et al. (2018)	Italy	Controlled trial	28	NR	49.47 (mean)	–			✔		
Davies et al. (2016)	United States	Follow‐up cohort	32	MMSE > 21	30–70	3–6.5		✔			
de La Torre et al. (2022)	Spain	RCT	60	RRMS	44.3 (mean)	–			✔		
Patrocinio de Oliveira et al. (2018)	Spain	Quasi experimental trial	52	McD, can walk ≥20 min	46.0 (mean)	–		✔			
Debolt and Mccubbin (2004)	Iran	Quasi experimental trial, case matched	37	Can walk ≥20 min without rest	NR	–		✔			✔
Dettmers et al. (2009)	Denmark	Controlled trial	30	From inpatient rehabilitation unit	45.8 (mean)	<4.5		✔	✔		✔
Eustis and Plummer (2022)	United States	Case report	1	Female SPMS	60	8		✔	✔		✔
Faramarzi et al. (2020)	Iran	RCT	89	Female, RRMS	18–50	–		✔			
Feys et al. (2013)	Netherlands	Quasi experimental trial	57	Education by the MS Society Flanders	NR	–		✔			✔
Filipi et al. (2010)	United States	Clinical trial	33	RRMS, SPMS, or PPMS, able to walk 25′	19–65	1–6.5		✔			✔
Fitzgerald et al. (2017)	United States	RCT secondary analysis	468	From BENEFIT study, CIS followed for conversion to MS, McD	NR	–	✔			✔	
Ford‐Johnson et al. (2016)	United States	RCT	18	CDMS	41.67 (mean)	–			✔		✔
Fox et al. (2012)	United States	Quasi experimental trial	45	RRMS, DMT ≥6 months at least two relapses during treatment	18–50	0–6.0		✔	✔	✔	✔
Fraser and Polito (2007)	United States	RCT	556	RRMS or PPMS	25–59				✔		
Fricska‐Nagy et al. (2016)	Hungary	Cross‐sectional	428	RRMS, glatiramer DMT > 1 year					✔		✔
Fruehwald et al. (2001)	Austria	Cross‐sectional	60	Poser criteria					✔		✔
Garrett et al. (2013a)	Ireland	RCT	121	0–2 mobility subscale, self‐referral	≥18			✔			✔
Garrett et al. (2013b)	Ireland	RCT follow up	314	NA	≥18			✔			✔
Gilbertson and Klatt (2017)	United States	Quasi experimental trial	25	NA	≥18				✔		✔
Gonsette et al. (2016)	England	RRMS or SPMS, ≥1 cerebral lesion	18	RRMS or SPMS, McD ≤15 years	18–55			✔		✔	✔
Goodman et al. (2009)	United States	RCT	301	Clinically defined MS	18–70			✔			
Graziano et al. (2014)	Italy	RCT	144	RRMS	20–65	1.0–5.5			✔		✔
Grazioli et al. (2019)	United States	Pilot RCT	20	No relapse within 1 month	25–55	2.5–5.5		✔			✔
Grech et al. (2015)	Australia	Cross‐sectional	108	RRMS or SPMS, McD, MMSE ≥25	≥18				✔		✔
Gudjonsdottir et al. (2021)	Iceland	Quasi experimental trial	41	Fampridine DMT during the first 16 months in Iceland	25–69			✔			
Gutierrez et al. (2005)	United States	Quasi experimental trial	8	Light physical activity for 3 months	46 (mean)	2.5–5.5		✔			✔
(Hajibabaei et al. (2020)	Iran	Quasi experimental trial	43	≥6 months, no psychological intervention	≥18				✔		✔
Hampson et al. (2020)	England	Quasi experimental trial	12	NA	25–81				✔		
Hansen et al. (2015)	Italy	Case‐matched RCT	37	McD	18–75	0.5–6.0		✔			
Hartung et al. (2002)	England	RCT	188	Step‐wise progression of disability	18–55	3.0–6.0		✔		✔	✔
Heine et al. (2017)	Netherlands	RCT	90	Severe fatigue, no relapse or corticosteroid < 3 months	18–70	≤6		✔			✔
Held Bradford et al. (2018)	United States	Case‐series	12	Self‐reported MS	≥18	Equiv. ≤6.5		✔			
Hogan et al. (2014)	Ireland	RCT	146	Diagnosis confirmed by physician	≥18	–		✔			✔
Holden and Isaac (2011)	England	Cross‐sectional	234	Self‐reported MS	18–65	–			✔		✔
Hoogs et al. (2011)	United States	Case‐matched quasi experimental trial	132	NA	46.4 (mean)	0–6.5			✔		✔
Hyphantis et al. (2008)	Greece	Case‐matched cross‐sectional	79	<12 months	21–59				✔		✔
Jelinek et al. (2016a)	Australia	Cross‐sectional	2312	Definite or possible MS	–	–	✔	✔			✔
Jelinek et al. (2016b)	Australia	Cross‐sectional	2469	Definite or possible MS	≥18	0–8^a^	✔	✔			
Kapoor et al. (2018)	England	RCT	889	Naïve to natalizumab DMT	18–58	3.0–6.5		✔			✔
Karadayi et al. (2014)	Turkey	Case‐matched cross‐sectional	31	McD	18–65	<5			✔		✔
Karimi et al. (2020)	Iran	Cross‐sectional	1025	≥6 months stable	20–62	–			✔		
Keller et al. (2021)	United States	Case‐matched clinical trial	14	RRMS, DMT, or > 6 months DMT	≥18	<4		✔			✔
Keser et al. (2011)	Turkey	Clinical trial	30	NA	–	1–5.5		✔	✔		✔
Khedr et al. (2022)	Egypt	Quasi experimental trial	110	NA	21–60				✔		
Kim et al. (2012)	United States	Cross‐sectional	694	Taking DMTs	≥18	–			✔		✔
Tepavcević et al. (2009)	Serbia	Cross‐sectional	109	McD	18–60	<8			✔		✔
Kolahkaj and Zargar (2015)	Iran	RCT	48	Females referred to Ahvaz	20–50	–			✔		
Kołtuniuk and Rosińczuk (2021)	Poland	Cross‐sectional	226	Treated with DMT	19–64	–			✔		
Kołtuniuk et al. (2021)	Poland	Cross‐sectional	109	RRMS with DMT	≥18	–			✔		✔
Kooshiar et al. (2015)	Iran	RCT	37	Females	19–45				✔		✔
Korostil and Feinstein (2007)	Canada	Cross‐sectional	140	NA	–	–			✔		✔
Kotterba et al. (2018)	Germany	Prospective observational	128	RRMS interferon beta‐1b DMT	≥18	≤ 5			✔		✔
Kuspinar et al. (2010)	Canada	Cross‐sectional	59	NA	–	–		✔			
Langeskov‐Christensen et al. (2022)	Denmark	RCT	86	From Danish MS clinics	–	–		✔			✔
Latinsky‐Ortiz (2022)	United States	Cross‐sectional and longitudinal observational	183	NA	20–64	–			✔		
Li et al. (2022)	United States	Cross‐sectional	748	Any type	≥18				✔		✔
Lincoln et al. (2020)	England	RCT	579	Cognitive problems	18–69	–			✔		✔
Lorefice et al. (2018)	Italy	Cross‐sectional	135	McD	–	–			✔		✔
Maier et al. (2016)	Romania	Cross‐sectional	351	RRMS or SPMS, McD, DMT	≥18	–			✔		✔
Mani et al. (2018)	Iran	Randomized trial	34	RRMS McD	20–45	–			✔		
Marck et al. (2018)	Australia	Longitudinal observational	95	NA	≥18	–	✔	✔			
Mark et al. (2008)	United States	Quasi experimental trial	5	PPMS or SPMS, McD, no relapse in 3 months, asymmetric upper limb motor deficit	–	–		✔			
Martinez‐Gonzlez (2015)	Spain	Case report	1	Female	19	–			✔		
Maurino et al. (2021)	Switzerland	Cross‐sectional	211	RRMS, McD	≥18	0–5			✔	✔	✔
Mauriz et al. (2013)	Spain	RCT	9	SPMS	–	>6.5	✔				
Mcguire et al. (2015)	United States	Controlled trial	72	McD	–	–			✔		✔
Meca‐Lallana et al. (2012)	Netherlands	Retrospective observational	68	RRMS, spasticity confirmed by scale in study	18–60	≤5.5			✔	✔	✔
Mekies et al. (2018)	France	Cross‐sectional	214	RRMS, DMT ≥6 months	–	–				✔	✔
Michalski et al. (2010)	Germany	Cross‐sectional	49	Outpatients department of neurology	–	3.3 (mean)			✔		✔
Mikula et al. (2021)	Slovakia	Cross‐sectional	165	McD	–	–			✔		✔
Mikukov et al. (2018)	Slovakia	Clinical trial	65	McD or Poser, MMSE > 23	≥18	1.5–8			✔		✔
Miller et al. (2020)	Netherlands	Prospective cross‐sectional	731	NA	–	–			✔		✔
Mirashrafi et al. (2021)	Iran	RCT	180	RRMS			✔				✔
Mohr et al. (2007)	United States	RCT	150	From the Kaiser Permanente medical care group	–	–			✔		✔
Montalban et al. (2011)	Spain	Cross‐sectional	281	RRMS	18–60	0–6			✔		✔
Moradi et al. (2015)	Iran	RCT	20	McD, males	20–55	1–6		✔			✔
Mostert (2002)	Switzerland	RCT	37	NA	–	–		✔			✔
Motl (2008)	United States	Cross‐sectional	133	From National MS Society	51.1 (mean)	–		✔	✔		✔
Motl et al. (2015)	United States	RCT	82	NA	18–64	–		✔			✔
Motl et al. (2013)	United States	Longitudinal observational	320	RRMS	–	–		✔			✔
Nag et al. (2022)	Australia	Longitudinal observational	774	Self‐reported MS	≥18	–			✔		✔
Nakazawa et al. (2018)	Japan	Cross‐sectional	65	NA	–	–			✔		✔
Naska et al. (2017)	Iran	RCT	60	Executive dysfunction	18–45	≤4			✔		
Nedeljkovic et al. (2016)	Serbia	RCT	37	RRMS, McD,	≥18	–		✔		✔	
Negaresh et al. (2019)	Iran	RCT	103	RRMS, McD, BMI 20–35 kg/m^2^	≥22	≤4		✔	✔		✔
Oz (2020)	Turkey	RCT	96	McD	18–65	–			✔		✔
Pahlavanzadeh et al. (2017)	Iran	RCT	70	Female ≥6 months, no relapses in previous month	–	0–5.5			✔		
Panda et al. (2018)	India	Cross‐sectional	90	McD	>18				✔		✔
Patti et al. (2011)	Italy	Prospective observational	331	RRMS, McD, no severe psychiatric disorder	18–50	≤4			✔		✔
Pavlikova et al. (2020)	Czech Republic	RCT	149	NA	–	–		✔			✔
Pawik et al. (2019)	Poland	Randomized trial	60	No contraindications	–	0–6		✔	✔		
Peterson (2001)	United States	Case report	1	Female	33	–		✔			
Plow et al. (2019)	United States	RCT	208	Physician confirmed	18–65	1–5^a^		✔			✔
Possa et al. (2017)	Italy	Cross‐sectional	38	Recent diagnosis (0–12 months)	–	–			✔		✔
Pouyanfard et al. (2020)	Iran	Controlled clinical trial	41	Referred to Shafa Hospital	18–50	–			✔		
Pust et al. (2021)	Germany	Cross‐sectional	608	Self‐reported	≥18	–			✔		✔
Rademacher (2021)	Germany	Secondary analysis pooled RCTs	130	RRMS, SPMS, McD	–	1–6.5			✔		
Rasova et al. (2006)	Czech Republic	Controlled clinical trial	112	No progression 3 months, relapse > 28 days, move independently	–	0–6.5			✔		✔
Razazian et al. (2016)	Iran	RCT	54	Females	25–50	–			✔		✔
Riemenschneider et al. (2022)	Denmark	RCT	84	RRMS ≤ 2 years	18–60	–		✔			
Rimmer et al. (2018)	United States	RCT	820	NA	18–70	0–7^a^		✔			✔
Rodgers et al. (1996)	United States	Quasi‐experimental	14	NA	33–65			✔	✔		✔
Romaniuc et al. (2020)	Romania	Observational prospective	349	Either RRMS or SPMS McD	≥18				✔		✔
Romberg et al. (2004)	Finland	Cross‐sectional	95	NA	31–54	1.0–5.5		✔			✔
Rooney et al. (2019)	Scotland	Cross‐sectional	412	NA	22–79	0–8^a^			✔		✔
Sandroff et al. (2019)	United States	Quasi‐experimental	32	Definite diagnosis, not completing adequate PA	18–64	4.0–6.0		✔	✔		
Sangelaji et al. (2014)	Iran	RCT	147	Relapse > 3 months, DMT	18–50	0–4.0		✔			✔
	Iran		97	RRMS		0–5		✔			✔
Senders et al. (2014)	United States	Cross‐sectional	119	McD, relapse > 90 days	18–90	≤8.0			✔		✔
Senders et al. (2019)	United States	RCT	59	McD, > 10 PSS, > 30 BDI or ≤26 MMSE	≥18	≤ 8			✔		✔
Sethy et al. (2010)	Netherlands	Case report	1	Male	34	3.5		✔			✔
Shahpouri et al. (2020a)	Iran	RCT	80	McD	≥18	≤5.5			✔		✔
Shahpouri et al. (2020b)	Iran	RCT	103	McD	≥18	≤5.5			✔		✔
Silva et al. (2018)	Brazil	Cross‐sectional	188	Surgery > 15 days	19–64		✔				✔
Skjerb'K et al. (2013)	Denmark	Randomized trial	19	Self‐reported	≥18	–		✔			✔
Socha et al. (2014)	Poland	Cross‐sectional	101	RRMS, McD	18–65	3.5 (median)	✔				
Stepleman et al. (2014)	United States	Retrospective audit	283	Positive depression screen	–	–			✔		
Stoeckel (2020)	United States	Cross‐sectional	112	Self‐reported MS	≥18	–		✔			✔
Tadic (2013)	Bosnia	Cross‐sectional	50	McD	18–69	<8			✔		✔
Tarakci et al. (2013)	Turkey	RCT	110	McD	–	2.0–6.5		✔			✔
Taspinar et al. (2015)	Turkey	RCT	36	RRMS	18–50	1.0–9.5		✔			✔
Taylor et al. (2006)	United States	Quasi‐experimental	9	NA	18–65			✔			✔
Timkova et al. (2021)	Slovakia	Cross‐sectional	162	NA	–	–			✔		✔
Titcomb et al. (2021)	United States	Randomized trial	77	RRMS, McD, FSS ≥4.0	–	–	✔				
Tollar et al. (2020)	United States	Randomized trial	82	Relapse frequency ≤1 over 5 years, BDI > 40	≥30	4.0–6.0		✔	✔		✔
Turner et al. (2019)	United States	RCT	64	MFIS ≥20	18–80	<6.5		✔			✔
Turner et al. (2016)	United States	Secondary analysis of RCT	64	NA	18–80	<6.5			✔		✔
van den Akker et al. (2018)	Netherlands	RCT	91	CIS20r ≥35, HADS > 11, ambulatory	–	≤ 6			✔		✔
van Kessel et al. (2008)	New Zealand	RCT	98	McD, ambulatory	–	≤ 6			✔		✔
Wallis et al. (2020)	Netherlands	Cross‐sectional	122	McD	≥18	–			✔		✔
Weiland et al. (2015)	Australia	Cross‐sectional	2138	NA	≥18	–	✔	✔			✔
Weinstein et al. (1999)	United States	RCT	251	RRMS, two relapses in 2 years	18–45	0–5			✔		
Weinstock‐Guttman et al. (2005)	United States	RCT	31	RRMS, stable > 2 months	18–60				✔		✔
Wilski (2016)	Poland	Cross‐sectional	210	No relapse > 30 days	–	–			✔		✔
Wilski et al. (2021)	Poland	Cross‐sectional	382	McD, no relapse > 30 days	–	–			✔		✔
Wilski et al. (2019)	Poland	Cross‐sectional	382	No relapse > 30 days	–	–			✔		✔
Wingo et al. (2020)	United States	Quasi‐experimental	20	RRMS, stable > 6 months, TICS‐m < 31	21–65	–	✔	✔	✔		✔
Yadav et al. (2016)	United States	RCT	61	RRMS McD < 15 years	18–70	≤ 6	✔	✔	✔		✔
Ytterberg et al. (2007)	Sweden	Longitudinal observational	83	DMT up to 24 months	–	–			✔	✔	✔
Ziemssen et al. (2016)	Germany	Prospective observational	754	NA	–	0–5		✔	✔		✔

Abbreviations: CIS20r, Checklist Individual Strength; DASS‐21, Depression Anxiety and Stress Scale‐21; DMT, disease‐modifying therapy; FSS, Fatigue Severity Scale; GHQ‐12, General Health Questionnaire 12‐item; HADS, Hospital Anxiety and Depression Scale; McD, McDonald criteria for MS; MFIS, Modified Fatigue Impact Scale; MMSE, Mini‐Mental State Examination; MS, multiple sclerosis; NA, not applicable; PPMS, primary progressive MS; RCT, randomized controlled trial; RRMS, relapsing‐remitting MS; SPMS, secondary progressive MS; T25FW, timed 25‐foot walk; TICS‐m, modified telephone interview for cognitive status.

**TABLE 2:** Ten most frequently used tools for nutrition studies^a^, classified into subcategories of tool type.

Tool	Times used^a^	Dietary assessment	Screening/Scoring	Biomarkers	Validation ref.
DHQ‐M	4		✔		Guan et al. (2022)
FFQ, non‐specific	3	✔			N/A
Blood test, non‐specific	3			✔	N/A
Urinary test, non‐specific	2			✔	N/A
CMP	1			✔	N/A
DII	1		✔		Wirth et al. (2017)^*^
EAT‐26	1		✔		Lane et al. (2004)^*^
Hs‐CRP	1			✔	N/A
TSH	1			✔	N/A
24‐h food recall	1	✔			Silveira et al. (2021)
All studies (total 12 tools)	20	3	3	6	

Abbreviations: CMP, comprehensive metabolic panel; DHQ, dietary habits questionnaire; DII, dietary inflammatory index; EAT‐26, Eating Attitudes Test; EDSS, Expanded Disability Status Scale; FFQ, Food Frequency Questionnaire; Hs‐CRP, high‐sensitivity C‐reactive protein; TSH, thyroid stimulating hormone.

^a^ Number of times the tool was used across the 166 studies included in the review.

* Not validated with a multiple sclerosis (MS) population.

For nutrition studies, only 12 tools were identified across 20 times of use in 14 studies (Table 3): Three tools were dietary assessment tools, three were screening or scoring tools, and six were biomarkers. The most frequently used dietary tool was the modified dietary habits questionnaire (DHQ), which was used four times across all studies. A majority of the tools were clinician‐reported, with 42% (*n* = 5) of the tools using patient‐reported outcomes via self‐administered measures from plwMS.

**TABLE 3:** Ten most frequently used tools for exercise studies^a^, classified into subcategories of tool type.

Tool	Times used^a^	Balance	Strength	Mobility	Endurance	Function	Coordination	Questionnaire	Validation ref.
6MWT	17			✔					Stellmann et al. (2015)
TUG	14			✔					Sebastião et al. (2016)
BBS	12	✔							Atteya et al. (2019)
T25FW	12			✔					Kalinowski et al. (2021)
GLTEQ	9							✔	Motl et al. (2018)
1RM	7		✔						Andreu‐Caravaca et al. (2020)
EXSE	7							✔	Resnick and Jenkins (2000)^*^
9HPT	6					✔			Feys et al. (2017)
IPAQ	6							✔	Fortune et al. (2019)
MSWS‐12	5							✔	Goldman et al. (2017)
All studies (total 55 tools)	156	4	9	16	8	1	1	16	

Abbreviations: 1RM, 1 Rep Max; 6MWT, 6‐minute walk test; 9HPT, 9 hole peg test; BBS, Berg Balance Scale; EXSE, exercise self‐efficacy scale; GLTEQ, Godin Leisure‐Time Exercise Questionnaire; IPAQ, International Physical Activity Questionnaire; MSWS‐12, 12‐item Multiple Sclerosis walking scale; T25FW, timed 25‐foot walk; TUG, timed up and go.

^a^ Number of times the tool was used across the 166 studies included in the review.

* Not validated with a multiple sclerosis (MS) population.

Fifty‐five exercise tools were reported a total of 156 times over 71 studies (Table 2), with 4 used to assess balance, 9 to assess strength, 16 to assess mobility, 8 for endurance, 1 for both function and coordination, and 16 were general physical activity questionnaires. The 6‐minute walk test (6MWT) was the most frequently used tool, reported 17 times. One quarter (*n* = 14) of the tools used to assess exercise were self‐administered tools and 70% (*n* = 39) of the tools used objective measures.

Across the six subcategories for psychology, there were 119 tools and 224 reported uses over 103 studies (Table 4). Five tools were used to assess personality, two to test intelligence, 20 behavioral assessments, five were projective, 32 for emotional intelligence tools, and 55 were neuropsychological tools. The most reported test for psychology was the Symbol Digits Modalities Test (SDMT) used 15 times. Unlike dietary and exercise interventions, self‐administered and subjective tools were more common for psychological studies. Self‐administered tools accounted for 62% (*n* = 78) of psychological tools and subjective tools accounted for 55% (*n* = 69).

**TABLE 4:** Ten most frequently used tools for psychology studies^a^, classified into subcategories of tool type.

Tool	Times used^a^	Personality	Intelligence	Behavioral	Projective	Emotional intelligence	Neuropsychological	Validation ref.
HADS	15						✔	Jerković et al. (2021)
SDMT	15						✔	Strober et al. (2019)
BDI‐II	13						✔	(Sacco et al., 2016)
PASAT	10						✔	Walker et al. (2021)
MMSE	6						✔	Beatty and Goodkin (1990)
STAI	6			✔				Santangelo et al. (2016)
DASS‐21	5			✔				Salehpoor and Hadianfard (2020)
HDRS	4			✔				Raimo et al. (2015)
BDI‐FS	3						✔	Benedict et al. (2003)
BRBN	3						✔	Neves et al. (2015)
All studies (122 tools)	224	5	2	20	5	32	55	

Abbreviations: BDI‐2, Beck Depression Inventory‐II; BDI‐FS, Beck Depression Inventory‐Fast Screen; BRBN, brief repeatable battery of neuropsychological tests; DASS‐21, Depression Anxiety and Stress Scale‐21; HADS, Hospital Anxiety and Depression Scale; MMSE, Mini‐Mental State Examination; PASAT, paced auditory serial addition test; SDMT, Symbol Digits Modalities Test; STAI, State Trait Anxiety Inventory.

^a^ Number of times the tool was used across the 166 studies included in the review.

**FIGURE 1:**
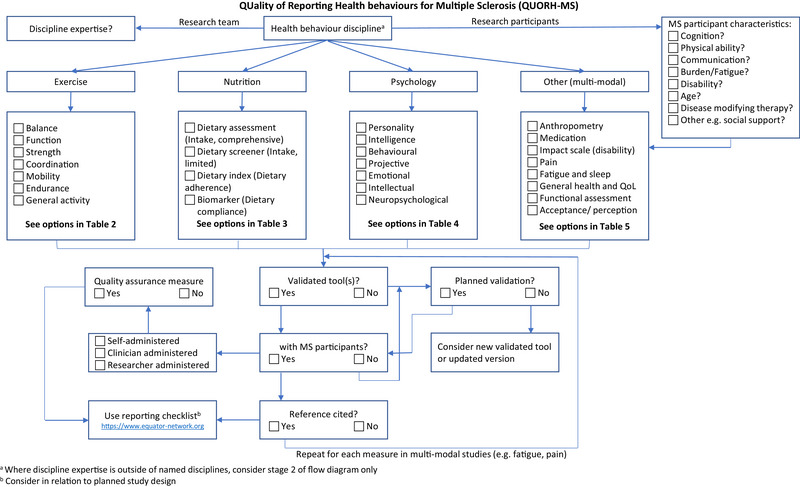
Checklist for multiple sclerosis (MS) lifestyle tool selection.

The use of validated tools was only addressed in 88 studies (53%) included in this review (Table S3). The tool selection aid (Figure 1) was intended to be based on the studies reported in this review and best practice research methodology from published reporting guides. A search of the EQUATOR network (https://www.equator‐network.org) was undertaken to identify suitable reporting checklists/aids with no tools available for quality improvement studies related to behavioral medicine, neurology, nutrition and dietetics, physiotherapy, or psychology. This same pattern was identified when limiting the search to reliability and agreement studies for each of the disciplines. When limited to reporting guidelines, only two were obtained in relation to neurology though neither was related to multiple sclerosis or its related disease classifications. The tool highlights a need for validation of all measures used in a study with respect to the population being targeted. In many instances, this is also in parallel with a need to consider quality assurance measures within the study and the need for references to validation studies to support statements made by the authors. The authors of this review also note a need for researchers to consider the difference between validity and reliability as some included studies of this review seemingly used the terms interchangeably.

## 4 DISCUSSION

The aim of this review was to identify the tools reported by researchers when implementing lifestyle interventions or health behavior studies for the management of MS. The outcomes of this review can inform health professionals about evidence‐based tools that may be used in practice, with the developed selection aid used to assist in choosing commonly known tool types. The results of this review indicated a wide variety of tools that can be used for lifestyle interventions for MS; however, it also highlights a collection of tools that are versatile as they are used across studies regardless of the intervention.

Exercise and psychology domains appeared to have a wide scope of investigation through the included studies, while nutrition appears to be less extensively studied with only 12% of the studies in this review focusing on nutrition interventions. This could be due to the emerging nature of nutrition and its role within MS management and an apparent focus on studies for the risk of MS rather than its management (Probst et al., 2022).

Exercise‐based interventions from this review indicate that general physical activity questionnaires and mobility assessments are the most varied yet frequently used forms of tools(*n* = 16 each). While questionnaires are useful, they often only assess activity levels to identify trends or are used in the evaluation of an intervention (van Poppel et al., 2010) and, therefore, are not an indicator of ability or capacity. However, mobility assessments were a common physical assessment administered by clinicians. This finding supports the priority of gait as a common concern among plwMS (Bethoux & Bennett, 2011).

For nutrition interventions, this review found that biomarkers were the most frequently used tool (*n* = 6). Biomarkers are indicative of intake and metabolism. They are recommended throughout the literature as they limit the errors that may occur when assessing intake, particularly for methods that rely on recall (Naska et al., 2017). Memory and cognitive impairments are common concerns for plwMS (Das Nair et al., 2016), and asking for a recall of information may be challenging or be restricted to the ability of a health practitioner to aid the recall using cognitive assistance such as prompts. Being able to accurately measure the food intake is important to reduce the risk of symptom‐related malnutrition or deficiency, though concerningly, we only identified three dietary assessments used in the included studies.

The results from this review indicate that psychological interventions mostly use neuropsychological tools. These tools assess broad areas of cognitive function and are commonly used with participants who have a health condition that affects the brain (Harvey, 2012). While the psychological implications including cognitive dysfunction are known for MS (Benedict et al., 2017), the application of cognitive tests like the SDMT is not consistent. Having a better understanding of the tools that can be used to assess cognition for MS will allow for a more accurate representation of the challenges plwMS may be experiencing.

A key finding outside of the number of tools used was the high variability in the use of a single tool. Sixteen tools were identified with multiple versions across the included studies. Tools with multiple versions include the Multiple Sclerosis Impact Scale (MSIS‐29), Beck Depression Inventory (BDI), General Health Questionnaire (GHQ), Fatigue Impact Scale (FIS), EuroQOL 5 Dimensions (EQ‐5D), the Multiple Sclerosis Self‐Management scale (MSSM), the Ashworth Scale (AS), Manual Muscle Testing (MMT), Toronto Alexithymia Scale (TAS), Coping Inventory for Stressful Situations (CISS), Coping Orientation for Problem Experiences (COPE), Treatment Satisfaction Questionnaire for Medication (TSQM), Fatigue Severity Scale (FFS), and Short form (SF‐36) and Minute Walking Tests. In this review, each was counted as one tool; however, an investigation into the differences between each version would assist in determining the differences and impact on the reported outcomes. While multiple versions of tools represent advances in a field, they may lead to confusion for health professionals who rely on evidence‐based outcomes to inform their practice. Having an understanding of which version is the most updated also ensures that researchers are informed during their study planning.

The tool selection aid that was informed by the included studies of this review will enable researchers to consider the implications of the study population characteristics, the context of the study, the expertise of the research team as well as the scientific rigor of the tools used to measure the outcomes. The tool considers the subdomains of nutrition, exercise, and psychology in planning for an intervention study and can be used in conjunction with the review findings reported in Tables 2, 3, 4 to reduce the variability of the tool types used and improve the consistency and comparability of study outcomes for MS.

A strength of this review was that this is the first attempt at summarizing assessment tools used for MS lifestyle interventions to guide researchers who are establishing themselves in this field of research. The review process followed best practices including multiple reviewers to ensure an accurate representation of the current evidence. The search strategy underwent various rounds of feasibility testing to ensure that the records retrieved were as extensive as possible. The members of the research team have discipline expertise across the investigated lifestyle areas (exercise and nutrition); however, the team did not include a member with psychology expertise. Therefore, the categorization of psychological tools relied on an investigation of the domain the tool assessed and definitions outlined in the *Handbook of Psychological Assessment* (Groth‐Marnat & Wright, 2016).

## 5 CONCLUSION

Evidence‐based practice for health is informed by research interventions and reviews. Evidence‐based practice allows health practitioners to improve the care offered to their clients to ensure that they are receiving best‐practice care to support behavior change and disease management (Asadoorian et al., 2010). This scoping review has given insight into the variability of tools used across the lifestyle interventions of nutrition, exercise, and psychology as well as additional tools that are considered to be multimodal. Greater consistency is needed for researchers to determine best‐practice tools within the disciplines of behavior change for MS so that health practitioners can use the evidence to advance their practice.

## AUTHOR CONTRIBUTIONS

**Yasmine Probst**: Conceptualization; methodology; data curation; validation; supervision; visualization; writing—original draft; writing—review and editing; formal analysis. **Emily Kinnane**: Data curation; investigation; formal analysis; writing—original draft.

## CONFLICT OF INTEREST STATEMENT

The lead author (Y.P.) of this review is a person living with multiple sclerosis and has received honoraria from Multiple Sclerosis Australia and Multiple Sclerosis Plus, funded research from Multiple Sclerosis Research Australia, and contributes to Multiple Sclerosis Australia advisory panels. E.K. does not have any conflicts to declare.

### PEER REVIEW

The peer review history for this article is available at https://publons.com/publon/10.1002/brb3.3635.

## Supporting information

Figure 1 PRISMA Flow diagram of literature search process.
Table 1 Search terms for key concepts.
Table 2 Discipline subcategories of tools, as well as the categories for the other tools used for symptom management of multiple sclerosis.
Table 3 Reporting of validated tools used in the included studies.

## Data Availability

All data of this review are available in scientific databases.
