# Supplementary material for: Quality of reporting health behaviors for multiple sclerosis (QuoRH‐MS): A scoping review to inform intervention planning and improve consistency of reporting
Source: Brain Behav. 2024 Aug 15;14(8):e3635. doi: 10.1002/brb3.3635 (PMC11327400; doi:10.1002/brb3.3635)
Supplement: Supplementary file 1 — Figure 1 PRISMA Flow diagram of literature search process. Table 1 Search terms for key concepts. Table 2 Discipline subcategories of tools, as well as the categories for the other tools used for symptom management of multiple sclerosis. Table 3 Reporting of validated tools used in the included studies. [file BRB3-14-e3635-s001.docx]

**Supplementary material**

**Figure 1:** PRISMA Flow diagram of literature search process.

**Table 1:** Search terms for key concepts.

| Key Concepts | Search Terms (searching with AND) |
| --- | --- |
| Multiple Sclerosis | “multiple sclerosis" OR MS  Multiple Sclerosis [Mesh] |
| Medication | medication OR medicine OR drug OR pharmaceutical OR pharmacology OR "disease modifying agents" OR patient* OR client* OR individual |
| Exercise | exercis* OR activit* OR scale OR tool OR intervention OR fitness OR movement OR training OR physical |
| Nutrition | diet OR nutrition OR "food intake" OR meals OR therap* OR scale OR measure OR tool OR intervention |
| Psychology | belief* OR attitude OR uptake OR stress OR psych* OR empower OR motivate OR enable OR therap* |

**Table 2:** Discipline subcategories of tools, as well as the categories for the other tools used for symptom management of multiple sclerosis.

| Nutrition | - Dietary assessment - Screening/Scoring | - Biomarkers - Questionnaire |
| --- | --- | --- |
| Exercise | - Balance - Strength - Mobility - Endurance | - Function - Coordination - Questionnaire |
| Psychology | - Personality - Intelligence - Behavioural | - Projective - Emotional intelligence - Neuropsychological |
| Other | - Medication - Anthropometric - Impact scale - Pain | - Fatigue and sleep - Health and Quality of life - Functional assessments - Acceptance and Perceptions |

**Table 3:** Reporting of validated tools used in the included studies.

| **Ref.** | **Tools** | **Validation**  **reported** |
| --- | --- | --- |
|  |  |  |
| (Agland et al., 2018) | DASS-21, sVAS, MusiQOL | **🗶** |
| (Alghwiri et al., 2020) | 6MWT, DGI, BBS, TUG, ABC, HGS, 9HPT, ST, SDMT, BDI, MFIS, PSQI, ESS, SF-36, Barthel Index | ✔ |
| (Amato et al., 2021) | TUG, Eye-hand reaction Test, Flamingo test, Wall squat Test, HGS, BREQ-3, EAT-26, MSSS, VAFS | **🗶** |
| (Anens et al., 2014) | PADS-R, EXSE, FES(S), ACTIVLIM questionnaire, FSS, MSIS-29 | ✔ |
| (Anens et al., 2017) | PADS-R, ACTIVLIM questionnaire, EXSE, FES(S), MSIS-29, FSS | **🗶** |
| (Artemiadis et al., 2012) | PSS, STAI, BDI, HLC Scale | ✔ |
| (Azimian et al., 2021) | BEST, PASAT, SDMT, EDSS | ✔ |
| (Sadeghi Bahmani et al., 2019) | BDI-FS, FSS, ISI | **🗶** |
| (Bakshi et al., 2000) | HDI, BDI, EDSS | **🗶** |
| (Banitalebi et al., 2020) | PCI | **🗶** |
| (Bassi et al., 2014) | BDI-2, PWBS, FQ, PANAS, SF-36, SWLS | ✔ |
| (Bijani et al., 2022) | Stress Management Questionnaire, General self-efficacy Scale, MSIS-29 | ✔ |
| (Bogosian et al., 2016) | EQ, SCS-SF, SEMCD, GHQ-12, AAQ-2 | **🗶** |
| (Boogar et al., 2018) | DASS-21, MMSE, MSQOL-54, SF-36 | ✔ |
| (Brenton and Goldman, 2016) | MSWS-12, BMI, MFIS | **🗶** |
| (Brenton et al., 2022) | 6MWT, Blood Test, SDMT, BDI, MFIS, MSFSS, MSQOL-54, EDSS, MSFC | **🗶** |
| (Canning and Hicks, 2020) | 1RM, T25FW | **🗶** |
| (Carletto et al., 2017) | BDI-II, BAI, PSS, M.I.N.I-Plus, FSS, B-IPQ, FAMS | ✔ |
| (Carvalho and Sá, 2012) | MSS, EDSS | **🗶** |
| (Cavalera et al., 2019) | HADS, MFIS, MSQOL-54 | ✔ |
| (Christopoulos et al., 2020) | TAS, GHQ-28 | ✔ |
| (Cohen et al., 2019) | T25FW, TUG, 2MWT, MSWS-12, BDI-II, BICAMS, FSMC | **🗶** |
| (Coote et al., 2017) | mCAFT, GLTEQ, EXSE, EGS, MOEES, EBBS, HADS, SDMT, MFIS, MSIS-29, EDSS | ✔ |
| (Crescentini et al., 2018) | BFI-44, STAI, BDI, FFMQ | **🗶** |
| (Davies et al., 2016) | 6MWT | **🗶** |
| (De La Torre et al., 2022) | WMS, SDMT, COWAT, PASAT | **🗶** |
| (Patrocinio De Oliveira et al., 2018) | CST, TUG, 1RM | **🗶** |
| (Debolt and Mccubbin, 2004) | MAS, TUG, EDSS | ✔ |
| (Dettmers et al., 2009) | Walking distance for time, BDI, MFIS, FSMC, HAQUAMS | **🗶** |
| (Eustis and Plummer, 2022) | EXSE, 5MWT, MSSS, MSIS-29, MFIS, PHQ-9 | ✔ |
| (Faramarzi et al., 2020) | 1RM, 6MWT, TUG | **🗶** |
| (Feys et al., 2013) | PASIPD, EXSE, MFIS, MSIS-29, SF-36, EDSS, PDDS | ✔ |
| (Filipi et al., 2010) | MFES, BBS, TUG, MFIS, MSFC | ✔ |
| (Fitzgerald et al., 2017) | MRI, Urine test (Sodium) | ✔ |
| (Ford-Johnson et al., 2016) | DVT, LNS, SDMT, CVLT-2, CMDI, STAI, MFQ, MFIS, FSS, MSQLI | ✔ |
| (Fox et al., 2012) | T25FW, 9HPT, PASAT, EDSS, MSFC | **🗶** |
| (Fraser and Polito, 2007) | MSSE |  |
| (Fricska-Nagy et al., 2016) | BDI, MSQOL-54, SF-36, FSS, FIS, MFIS | ✔ |
| (Fruehwald et al., 2001) | MMSE, ZDRS, ZARS, Zerssen Emotional State Scale, QOL index, EDSS | **🗶** |
| (Garrett et al., 2013a) | 6MWT, MFIS, MSIS-29 | ✔ |
| (Garrett et al., 2013b) | 6MWT, MSIS-29v2, MFIS |  |
| (Gilbertson and Klatt, 2017) | MHI, FFMQ, MFIS, SF-36 | ✔ |
| (Gonsette et al., 2016) | MRI, T25FW, 9HPT, EDSS | **🗶** |
| (Goodman et al., 2009) | T25FW, AS, MSWS-12, LEMMT | **🗶** |
| (Graziano et al., 2014) | PANAS, CES-D, Identity Motives Scale, MSQOL-54 | ✔ |
| (Grazioli et al., 2019) | BBS, TUG, 6MWT, 10MWT, FSS, MSQOL-54, PHQ-9 | **🗶** |
| (Grech et al., 2015) | BDI, IGT, BADS, MSET, WLG, Reading Span Test, Test of Everyday Attention, Hayling Sentence Completion Test, TMT, SDMT, DHS, STAI, MSQOL-54 | ✔ |
| (Gudjonsdottir et al., 2021) | T25FW, MSWS-12 | **🗶** |
| (Gutierrez et al., 2005) | 3-minute stepping Test, 1RM, MFIS | **🗶** |
| (Hajibabaei et al., 2020) | MLQ, MSQOL-54 | ✔ |
| (Hampson et al., 2020) | HADS | **🗶** |
| (Hansen et al., 2015) | PASIPD | **🗶** |
| (Hartung et al., 2002) | MRI, AI, EDSS | **🗶** |
| (Heine et al., 2017) | CPET, MFIS, FSS, CIS20r, IPA | **🗶** |
| (Held Bradford et al., 2018) | SQUASH, ABC, CARE | ✔ |
| (Hogan et al., 2014) | BBS, 6MWT, MSIS-29, MFIS | ✔ |
| (Holden and Isaac, 2011) | 9-item mood subscale of CMDI, SF-36, SF-MPQ | ✔ |
| (Hoogs et al., 2011) | MACFIMS, COWAT, JLO, CVLT2, BVMTR, SDMT, PASAT, DKEFS, Sorting test, BDI-FS, FSS, SIP | ✔ |
| (Hyphantis et al., 2008) | Symptom Distress Checklist-90-R, DSQ, HDHQ, MMPI Ego Strength, GHQ-28 | **🗶** |
| (Jelinek et al., 2016a) | IPAQ, DHQ, SCQ, MSQOL-54, PDDS | ✔ |
| (Jelinek et al., 2016b) | IPAQ, DHQ-modified | ✔ |
| (Kapoor et al., 2018) | T25FW, 9HPT, EDSS | **🗶** |
| (Karadayi et al., 2014) | SCID-1, HDRS, HARS, MMSE, FSS, EDSS, GAF | ✔ |
| (Karimi et al., 2020) | DASS-21 | ✔ |
| (Keller et al., 2021) | GLTEQ, T25FW, 6MWT, MFIS | ✔ |
| (Keser et al., 2011) | BBS, HAD, SF-36, EDSS, MSFC | **🗶** |
| (Khedr et al., 2022) | PSS, POMS | ✔ |
| (Kim et al., 2012) | BDI-II, ISS | ✔ |
| (Tepavcević et al., 2009) | HDRS, MMS MSQOL-54, EDSS | ✔ |
| (Kolahkaj and Zargar, 2015) | DASS-21 | ✔ |
| (Kołtuniuk and Rosińczuk, 2021) | BDI, HADS-M | **🗶** |
| (Kołtuniuk et al., 2021) | PSS-10, Mini-COPE, MusiQOL | **🗶** |
| (Kooshiar et al., 2015) | MFIS, FSS, MQLI | ✔ |
| (Korostil and Feinstein, 2007) | SCID-IV, BSS, HADS, SSSI, EDSS | **🗶** |
| (Kotterba et al., 2018) | HADS, MFIS, PSQI, SF-36, ESS, HPAL, EDSS | **🗶** |
| (Kuspinar et al., 2010) | Modified Canadian Aerobic Fitness Test, 6MWT, Vertical Jump Test, Push up test, Partial curl ups, HGS | ✔ |
| (Langeskov-Christensen et al., 2022) | SSST, MSWS-12, Incremental exercise test, 6MWT, SF-36, MFIS, FSS | ✔ |
| (Latinsky-Ortiz, 2022) | SPWB, CHQ | **🗶** |
| (Li, 2022) | PAWE, PROMIS, BRS, UW-SES, PSS, Neuro-QOL, PDDS | ✔ |
| (Lincoln, 2020) | MSNQ, BRBN, EMQ-p, Doors and People, Trail Making Test, FSS, EQ-5D-5L, GNDS, MSIS-29 | **🗶** |
| (Lorefice, 2018) | COPE-NVI, EDSS | ✔ |
| (Maier, 2016) | BDI-II, EDSS | **🗶** |
| (Mani, 2018) | ACE, CPT, WCST, BRIEF-A, MFQ, WMS-R | ✔ |
| (Marck, 2018) | IPAQ short form, DHQ-modified | ✔ |
| (Mark, 2008) | 30-item MAL, WMFT | ✔ |
| (Martinez-Gonzlez, 2015) | BRBN for MS, WAIS, PASAT, TMT, ROCF, RAVLT, Phonemic, FAS, 16PF-5, ST | ✔ |
| (Maurino, 2021) | SMSS, SDMT, EDSS | ✔ |
| (Mauriz, 2013) | Blood test, Urine test | **🗶** |
| (Mcguire, 2015) | MSNQ, BDI, MHI, PSS, MFIS, PES, PDQ | **🗶** |
| (Meca-Lallana, 2012) | PSFS, MAS, ATRS, MSQOL-54, Global Pain Scores, EDSS | **🗶** |
| (Mekies, 2018) | TSQM, EQ-5D-3L | **🗶** |
| (Michalski, 2010) | HADS, SF-36, EDSS | **🗶** |
| (Mikula, 2021) | RSE, GHQ-28, MSSM | **🗶** |
| (Mikukov 2018) | BDI, MMSE, MFIS, EDSS | **🗶** |
| (Miller, 2020) | HADS, MAAS, IIRS | ✔ |
| (Mirashrafi, 2021) | hs-CRP, BMI, %Body Fat | ✔ |
| (Mohr, 2007) | BDI-II, FIS, GNDS | ✔ |
| (Montalban, 2011) | BDI-II, HAQUAMS | ✔ |
| (Moradi, 2015) | 10TW, 1RM, Flamingo Stand Test, EDSS | ✔ |
| (Mostert, 2002) | AS, BPAQ, SF-36, FSS, EDSS | ✔ |
| (Motl, 2008) | GLTEQ, MSSE, MSIS-29 | ✔ |
| (Motl, 2015) | GLTEQ, IPAQ, BMI, PDDS | ✔ |
| (Motl, 2013) | GLTEQ, MSSE | ✔ |
| (Nag, 2022) | PMS, FSS, PHQ-9 | ✔ |
| (Nakazawa, 2018) | BDI-II, RS, MSQOL-54 | ✔ |
| (Naska, 2017) | WCST, PASAT, HARS | **🗶** |
| (Nedeljkovic et al., 2016) | EDSS, FIM,  BDI, MSQoL-54, SF-36 | **🗶** |
| (Negaresh, 2019) | TUG, BDI, FSS, EDSS | ✔ |
| (Oz, 2020) | WCI, MSQOL-54, BSI | ✔ |
| (Pahlavanzadeh, 2017) | DASS-21 | ✔ |
| (Panda, 2018) | HADS, BDI, MMSE, EDSS, GHQ-12  AUDIT | ✔ |
| (Patti, 2011) | BRB, ST, HDRS, FIS, MSQOL-54 | **🗶** |
| (Pavlikova, 2020) | BBS, TUG, EDSS | **🗶** |
| (Pawik, 2019) | RMI, PGWBI, HADS | **🗶** |
| (Peterson, 2001) | MMT | **🗶** |
| (Plow, 2019) | GLTEQ, MSIS-29, FIS | **🗶** |
| (Possa, 2017) | BDI-II, STAI, QPF-R, MOCQ-R, IES-R, PMQ, PSI, FSS, MSQOL-54 | **🗶** |
| (Pouyanfard, 2020) | BDI-II, BAI, MHS | ✔ |
| (Pust, 2021) | BDI-II, TAS-26, CTQ, SMI, PDDS | ✔ |
| (Rademacher, 2021) | BICAMS, SDMT, VLMT, BVMT-R | **🗶** |
| (Rasova, 2006) | BDI, MFIS, SF-MPQ, EDSS | **🗶** |
| (Razazian, 2016) | BDI, FSS | ✔ |
| (Riemenschneider, 2022) | Incremental Exercise Test, BPAQ | **🗶** |
| (Rimmer, 2018) | GLTEQ, BBS, TUG, T25FW, 6MWT, 5STS, EXSE, MOEES, EGS, SF-36, MFIS | **🗶** |
| (Rodgers, 1996) | HGS, Word List learning and memory, SDMT, BDI, SILS | **🗶** |
| (Romaniuc, 2020) | BDI-II, EDSS | **🗶** |
| (Romberg, 2004) | Incremental Exercise Test, EDSS | **🗶** |
| (Rooney, 2019) | HADS, MSNSQ, FSS, MSIS-29, PSQI | ✔ |
| (Sandroff, 2019) | T25FW, 6MWT, SDMT, PASAT | ✔ |
| (Sangelaji, 2014) | BBS, 6MWT, FSS, Mental QOL, EDSS, Physical QOL | **🗶** |
|  | 1RM, BBS, TUG, 10MWT, 6MWT, BMI, Family support service, |  |
| (Senders, 2014) | PSS, FFMQ, CD-RISC, BRIEF-COPE, SF-36 | ✔ |
| (Senders, 2019) | PSS, CD-RISC, PASAT, CEQ, SF-36 | ✔ |
| (Sethy, 2010) | MMT, 14MW, TUG, 6MWT, MFIS, EDSS | ✔ |
| (Shahpouri, 2020a) | AMT, PRMQ, EMQ, BDI, MSQOL-54 | **🗶** |
| (Shahpouri, 2020b) | EMQ, AMT, PRMQ, BDI, DGS, MSQOL-54 | ✔ |
| (Silva, 2018) | FFQ, DII, EDSS | ✔ |
| (Skjerb‘K, 2013) | 5STS, MSFC, VAS (common symptoms) | ✔ |
| (Socha, 2014) | FFQ | **🗶** |
| (Stepleman, 2014) | BDI-FS | ✔ |
| (Stoeckel, 2020) | EXSE, GLTEQ, PDDS | ✔ |
| (Tadic, 2013) | HDRS, HARS, SF-36, MSQOL-54, EDSS | ✔ |
| (Tarakci, 2013) | BBS, 10MW, MAS, MSQOL-54 | ✔ |
| (Taspinar, 2015) | BBS, 10MW, EDSS | ✔ |
| (Taylor, 2006) | 1RM, 2MWT, Timed Stair Walk, MSIS-29 |  |
| (Timkova, 2021) | B-IPQ, RSE, PSQI, GHQ-28 | **🗶** |
| (Titcomb, 2021) | WFR | **🗶** |
| (Tollar, 2020) | 6MWT, BDI, MSIS-29, EQ5D | ✔ |
| (Turner, 2019) | GLTEQ, MFIS, PHQ-9 | ✔ |
| (Turner, 2016) | BADS-Activation, PHQ-9 | ✔ |
| (Van Den Akker, 2018) | ICQ, GSES, CISS21, FPQ, BIPQ, HADS, CIS20r, SF-36 | ✔ |
| (Van Kessel, 2008) | HADS, PSS, Brief IPQ, ESS, Sleep Problems Questionnaire, EDSS, Fatigue Scale, | ✔ |
| (Wallis, 2020) | HADS-A, HADS-D, CFQ, GOT, COWAT, CVLT-2, WMS, FSS | ✔ |
| (Weiland, 2015) | IPAQ, DHQ-modified, BMI | ✔ |
| (Weinstein, 1999) | BRBN, PASAT, SDMT, BSRT, SPART, WLG | **🗶** |
| (Weinstock-Guttman, 2005) | MHI, MFIS, SF-36, PCS, EDSS | **🗶** |
| (Wilski, 2016) | BIPQ, RSE, GSES, EDSS, MSSM-R, TBS | ✔ |
| (Wilski, 2021) | CISS, MSSM-R, MSIS-29 | ✔ |
| (Wilski, 2019) | CISS, MSIS-29, AIS | ✔ |
| (Wingo, 2020) | T25FW, 9HPT, 24-hour food recall, SDMT, GAD-7, PHQ-9, SF-36, MSFC | **🗶** |
| (Yadav, 2016) | RAPA, CBC, CMP, TSH, vitamin B12 status, Blood test, FFQ, BDI, FSS, MSQLI, EDSS, MSFC | ✔ |
| (Ytterberg, 2007) | MSIS-29, MSFC, SDMT, BDI, MSIS-29, MSFC | **🗶** |
| (Ziemssen, 2016) | T25FW, 9HPT, PASAT, MUSIC, General depression scale, FSMC, EDSS, FAMS, MSFC | **🗶** |
